# Histological and Transcriptomic Insights into Rugose Surface Formation in Pepper (*Capsicum annuum* L.) Fruit

**DOI:** 10.3390/plants14152451

**Published:** 2025-08-07

**Authors:** Yiqi Xie, Haizhou Zhang, Chengshuang Li, Qing Cheng, Liang Sun, Huolin Shen

**Affiliations:** 1 Department of Vegetable Science, College of Horticulture, China Agricultural University, Beijing 100193, China; xieyiqi277@163.com (Y.X.); zhz1999123@163.com (H.Z.); 13309045006@163.com (C.L.); chengqing2020@cau.edu.cn (Q.C.); 2 Sanya Institute, China Agricultural University, Sanya 572025, China

**Keywords:** pepper, fruit surface rugosity, epidermal morphology, comparative transcriptome analysis, fruit texture

## Abstract

The rugose surface trait in pepper (*Capsicum annuum* L.), marked by ridges and depressions on the fruit epidermis, is linked to improved fruit texture. To investigate its regulatory basis, histological, textural, and transcriptomic differences, contrasting genotypes were analyzed. Histological analysis revealed that disorganized epidermal cell layers contribute to rugosity, with morphological differences emerging around 10 days post-anthesis (DPA). A computer-aided design (CAD)-based rugosity index (RI) was developed and showed strong correlation with sensory rugosity scores (R^2^ = 0.659, *p* < 0.001). Texture analysis demonstrated that increasing surface rugosity was associated with reduced rupture force and hardness, as well as elevated pectinase activity. Comparative transcriptome profiling identified 10 differentially expressed genes (DEGs) related to microtubule dynamics (e.g., *CA03g18310* and *CA09g13510*) and phytohormone signaling (e.g., *CA03g35180* and *CA08g12070*), which exhibited distinct spatial and temporal expression patterns. These findings suggest that coordinated cytoskeletal remodeling and hormonal regulation drive epidermal disorganization, leading to surface rugosity and altered fruit texture. The study provides novel insights into the molecular basis of fruit surface morphology and identifies promising targets for breeding high-quality pepper cultivars.

## 1. Introduction

Fruit quality is a crucial aspect in horticultural crop cultivation, encompassing appearance, nutritional, and sensory qualities and directly impacting marketability and consumer acceptance. As a key attribute of sensory quality in horticultural crops, texture play a critical role in shaping consumer perception of fruit quality [1,2,3,4,5]. While breeders have traditionally focused on improving agronomical important traits, such as stress resistance and nutritional content [6,7,8,9,10,11], sensory qualities of the fruit, particularly texture, have often been overlooked.

Pepper (*Capsicum annuum* L.), a major crop in the Solanaceae family, is widely grown for both vegetable and spice use. It is mainly produced in China, Mexico, Türkiye, and Indonesia. Due to its diverse agronomic traits, pepper has become a key focus in breeding programs. In 2023, global production reached 38.31 million tons, underscoring its importance in global agriculture [12,13]. One notable texture-related trait is the rugose surface, characterized by an uneven fruit surface. Peppers with a rugose surface are particularly favored by consumers for their crisp and tender texture, suggesting that this phenotype could be leveraged to enhance fruit quality. Despite consumer preference for rugose surface peppers due to their crisp texture, the physiological and genetic mechanisms underlying this trait remain poorly understood. Fruit texture is inherently complex, involving properties such as hardness, rupture force, chewiness, cell wall composition, pectinase activity, and cell structure [5,14,15,16,17,18]. Texture Profile Analysis (TPA) is a widely used technique to simulate the sensory texture of the pulp by mimicking the chewing process [18,19,20]. The major components of the cell wall, including cellulose, hemicellulose, and pectin, are pivotal in determining fruit texture [21], with pectin playing a key role in cell wall stability and fruit firmness [22,23,24]. Pectin degradation is commonly associated with fruit softening [25,26], and cell shape and size can further influence texture through their impact on cell-to-cell adhesion [17,18,27].

As a specialized surface phenotype, rugose texture may be governed by mechanisms similar to those regulating other fruit surface traits such as trichomes and cuticles. Recent advances have highlighted the importance of fruit surface traits, such as trichomes and cuticles, in determining fruit quality [28,29,30,31,32]. Surface traits are known to confer benefits like resistance to pests, reduced water loss, and improved appearance, making them economically significant [31,33,34,35]. Although studies in horticulture crops like cucumber and apple have identified key genes regulating surface features [31,36], the genetic and physiological bases of rugose surface formation in pepper remain underexplored.

To address the current knowledge gap in understanding the biological basis of fruit surface rugosity in pepper, this study aimed to (i) quantitatively characterize surface rugosity using a CAD-based method and evaluate its relationship with fruit texture traits; (ii) investigate the anatomical basis of rugose surface formation through histological analysis; and (iii) identify candidate genes and molecular pathways associated with rugosity through comparative transcriptome profiling. Together, these efforts seek to uncover the cellular and genetic mechanisms underlying surface morphology and provide molecular targets for improving fruit texture in pepper breeding.

## 2. Results

### 2.1. Validation of the CAD-Based Rugosity Quantification Method

Linear regression analysis revealed a strong correlation between the CAD-derived rugosity index (RI) and manual sensory evaluations (R^2^ = 0.659, *p* < 0.001, Figure S1), indicating that the RI accounts for approximately 65.9% of the variance in visual classification of surface roughness. These findings confirm the reliability of the CAD-based method for objective quantification of fruit surface rugosity in pepper. To facilitate subsequent analysis of its relationship with texture traits, the F_2_ population was grouped into four rugosity levels based on RI values: Level 1: RI 0–1.5%; Level 2: 1.5–2.5%; Level 3: 2.5–3.5%; and Level 4: >3.5%. This classification system was used in further analyses to assess the association between surface rugosity and fruit texture characteristics.

### 2.2. Association Between Fruit Surface Rugosity and Texture-Related Indicators

**Figure 1:**
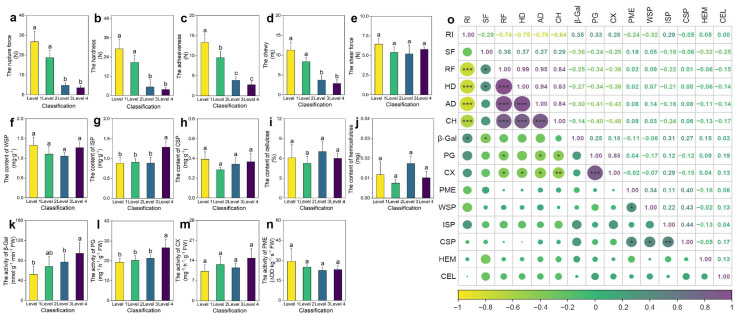
Differences in pepper fruit texture (**a**–**e**), cell wall components (**f**–**j**), and pectinase activity (**k**–**n**) across different levels of rugose surface classification, along with correlations among the indicators. (**a**) Rupture force (RF). (**b**) Hardness (HD). (**c**) Adhesiveness (AD). (**d**) Chewiness (CH). (**e**) Shear force (SF). (**f**) Water-soluble pectin (WSP) content. (**g**) Ionically soluble pectin (ISP) content. (**h**) Covalently soluble pectin (CSP) content. (**i**) Cellulose (CEL) content. (**j**) Hemicellulose (HEM) content. (**k**) β-Galactosidase (β-Gal) activity. (**l**) Polygalacturonase (PG) activity. (**m**) Cellulase (CX) activity. (**n**) Pectin methylesterase (PME) activity. (**o**) Correlation analysis among all indicators. Data are presented as the mean ± SE (*n* = 3). The size of the points indicates the strength of the correlation (absolute Pearson’s r), with larger points representing stronger correlations. For (**a**–**n**), different letters indicate statistically significant differences (*p* < 0.05). For (**o**), * denotes significance at *p* < 0.05, ** at *p* < 0.01, and *** at *p* < 0.001.

Fruit surface rugosity exhibited a significant impact on multiple textural attributes. As RI increased from Level 1 to Level 4, a progressive reduction in rupture force, hardness, adhesiveness, and chewiness was observed (Figure 1a–d). Specifically, rupture force in smooth fruits (Level 1) was 6.55 times higher than in highly rugose fruits (Level 4), indicating markedly reduced mechanical integrity in the latter. In contrast, shear force remained unchanged across rugosity levels, suggesting that it is not directly influenced by surface morphology (Figure 1e).

To elucidate the underlying biochemical basis of these textural differences, the content of major cell wall polysaccharides and the activity of key modifying enzymes were quantified. Among pectin fractions, ionically soluble pectin (ISP) was significantly elevated in Level 4 fruits (*p* < 0.05; Figure 1g), suggesting enhanced pectin solubilization. Furthermore, the activities of β-galactosidase (β-GAL) and polygalacturonase (PG), two enzymes implicated in pectin deconstruction and middle lamella dissolution, were significantly higher in highly rugose fruits (*p* < 0.05; Figure 1k,l). This enzymatic enhancement is consistent with increased pectin turnover and cell wall softening.

Correlation analysis (Figure 1o) revealed significant negative correlations between rugosity and rupture force, hardness, adhesiveness, and chewiness, whereas rugosity positively correlated with β-GAL and PG activities. These findings indicate that increased surface rugosity promotes enzymatic degradation of pectin-rich domains. This results in weakened intercellular adhesion and reduced tissue rigidity, thereby enhancing perceived crispness and tenderness in rugose fruits. The rugosity trait thus integrates morphological and biochemical modifications to shape fruit textural quality.

### 2.3. Morphological and Cellular Analyses

**Figure 2:**
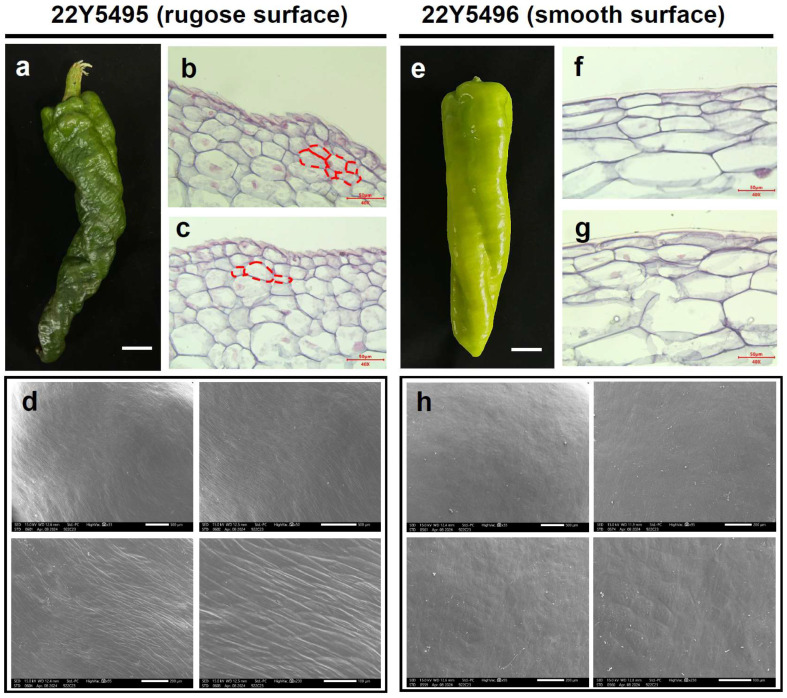
Morphological characteristics of rugose and smooth surface pepper fruits. (**a**) Pepper fruits from the rugose surface parent “22Y5495” at 30 days post-anthesis. (**b**,**c**) The longitudinal section morphological characteristics of rugose surface fruits in the main stage. The red dashed lines indicate the cell layers implicated in the formation of rugose surface. (**d**) SEM images of rugose surface pepper fruit. (**e**) Pepper fruits from the smooth surface parent “22Y5496”. (**f**,**g**) The longitudinal section morphological characteristics of smooth surface fruits in the main stage. (**h**) SEM images of smooth surface fruit.

To determine whether the rugose phenotype is associated with systemic developmental abnormalities, we compared whole-plant architecture and vegetative organ morphology between the parental lines. No obvious differences in stem thickness, leaf morphology, flower structure, or plant stature were observed (Figure S2). Pollen viability and seed set were also comparable, suggesting that the rugose phenotype is restricted to fruit epidermal development. Morphological differences between smooth and rugose pepper fruits were examined using paraffin sectioning and scanning electron microscopy (SEM). In the smooth fruit line (22Y5496), epidermal cells were arranged in a regular and orderly pattern, contributing to a flat and even fruit surface (Figure 2e–g). In contrast, the protruding regions of rugose fruits (22Y5495) exhibited disorganized epidermal cell layers, resulting in surface folding and ridge formation (Figure 2a–c). Furthermore, rugose fruits displayed a thinner waxy cuticle, and their epidermal cells were more exposed and arranged in an undulating pattern (Figure 2d,h). These structural deviations may reflect underlying alterations in epidermal cell proliferation dynamics and growth polarity. Specifically, the observed disorganization may arise from disrupted regulation of periclinal and anticlinal cell division, possibly driven by spatially heterogeneous phytohormone distribution during early fruit development. Such developmental modifications are likely to contribute to localized tissue bulging, leading to the characteristic rugose surface phenotype.

### 2.4. Transcriptome Analyses and Validation of Developmental Stages in Rugose Fruits

RNA-seq analysis was performed on fruits collected from two parental lines with contrasting epidermal phenotypes at three key developmental stages. Samples were designated as M6 (rugose, 6 days post-anthesis (DPA)), M10 (rugose, 10 DPA), M14 (rugose, 14 DPA), G6 (smooth, 6 DPA), G10 (smooth, 10 DPA), and G14 (smooth, 14 DPA). A total of 119.82 Gb of clean data was generated, with an average of 6.66 Gb per sample (Table S1). A total of 802,638,682 high-quality reads was generated. An average of 81% reads were uniquely mapped (Table S2). Principal component analysis (PCA) demonstrated a clear separation between rugose and smooth fruit samples along the first principal component (PC1, explaining 29.38% of the variance; Figure S3b), indicating distinct transcriptional programs associated with surface phenotype.

Gene Ontology (GO) enrichment analysis of differentially expressed genes (DEGs) across three developmental comparisons (G6 vs. M6, G10 vs. M10, and G14 vs. M14) revealed common enrichment of cellular component (CC) terms related to the nucleus, nucleosome, microtubules, and cytoskeleton. Given their fundamental role in cell shape maintenance, intracellular transport, and division [37,38], microtubules were of particular interest. The enrichment of molecular function (MF) terms such as “microtubule motor activity” and “microtubule binding” further suggested the involvement of dynamic microtubule remodeling in regulating epidermal architecture and surface morphology (Figure S3d).

**Figure 3:**
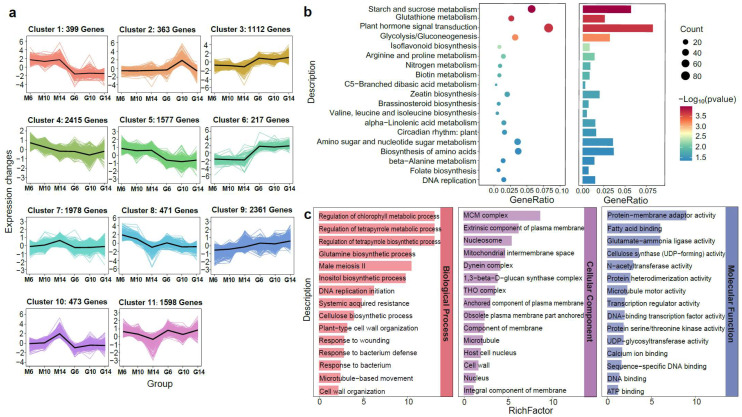
Gene expression and KEGG enrichment analyses during the development of rugose surface and smooth surface pepper fruits based on their accumulation patterns. (**a**) K-means clustering analysis of gene expression patterns. The *x*-axis represents the different samples collected from two parental lines (M: rugose; G: smooth) at three developmental stages (6, 10, and 14 days post-anthesis). The *y*-axis represents the standardized genes expression. The colored areas in the background represent the expression trajectories of individual genes within each cluster, while the bold black line denotes the average expression profile of all genes in the cluster. (**b**) KEGG pathway enrichment analysis of differentially expressed genes (DEGs) for main cluster based on K-means clustering. (**c**) GO enrichment analysis of DEGs for main cluster based on K-means clustering.

To explore stage-specific transcriptional dynamics, K-means clustering was performed on DEGs from six developmental comparisons (G6 vs. M6, G10 vs. M10, G14 vs. M14, M6 vs. M10, M10 vs. M14, and M6 vs. M14), resulting in 11 co-expression modules (Figure 3a). Clusters 1, 5, and 10 showed predominant expression in rugose fruits, while Clusters 3 and 6 were mainly expressed in smooth fruits. KEGG enrichment analysis revealed these modules were linked to pathways involved in cell wall biosynthesis, cell expansion, hormone signaling, and stress response, including starch and sucrose metabolism and alpha-linolenic acid metabolism (Figure 3b). These findings suggest that rugosity formation is regulated by coordinated modulation of cell wall remodeling and phytohormone signaling. GO enrichment analysis further confirmed the significance of microtubule-related functions (microtubule-based movement, microtubule structure, and motor activity) in the BP, CC, and MF categories (Figure 3c), underscoring their potential roles in surface morphology differences between rugose and smooth fruits.

**Figure 4:**
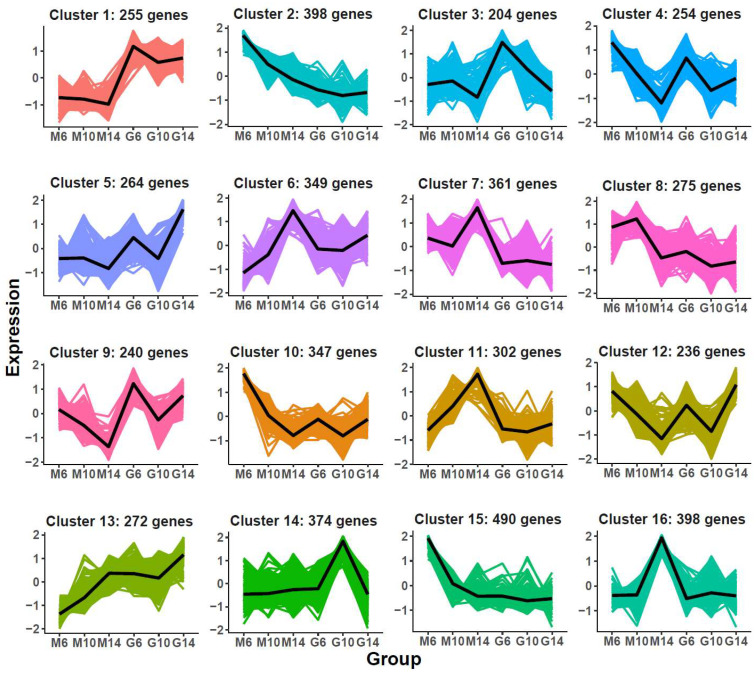
K-means clustering of differentially expressed genes (DEGs) identified as specific to the developmental process of rugose fruit. The *x*-axis represents different samples collected from two parental lines (M: rugose; G: smooth) at three developmental stages (6, 10, and 14 days post-anthesis). The *y*-axis denotes the standardized gene expression levels. The colored areas in the background represent the expression trajectories of individual genes with-in each cluster, while the bold black line denotes the average expression profile of all genes in the cluster.

To further dissect regulatory mechanisms specific to rugose fruit development, K-means clustering was applied to 5019 DEGs unique to the rugose line, yielding 16 co-expression modules (Figure 4; Table S3). Genes in Cluster 1 were consistently highly expressed in smooth fruits, while Clusters 7 and 11 displayed elevated expression in rugose fruits, particularly at 14 DPA (M14).

**Figure 5:**
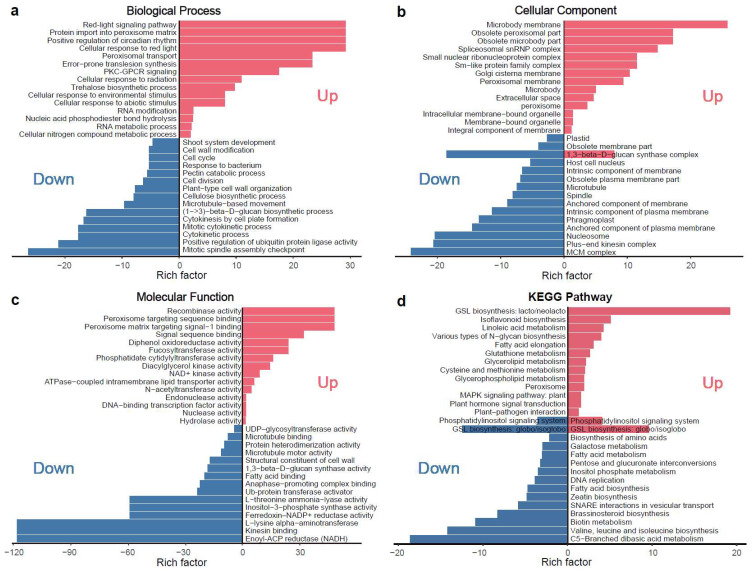
KEGG pathway and GO enrichment analysis of differentially expressed genes (DEGs) for main cluster based on K-means clustering. (**a**) Enrichment results for the biological process (BP) category in the GO enrichment analysis. (**b**) Enrichment results for the cellular component (CC) category in the GO enrichment analysis. (**c**) Enrichment results for the molecular function (MF) category in the GO enrichment analysis. (**d**) KEGG enrichment pathway analysis. The red color indicates the enrichment results of upregulated genes, while the blue color indicates the enrichment results of downregulated genes.

Enrichment analyses highlighted significant involvement of microtubule-related genes, including kinesin-like proteins, in rugosity-associated morphological changes (Figure 5a–c; Table S4). KEGG pathway enrichment further identified key metabolic pathways, including plant hormone signal transduction, brassinosteroid biosynthesis, and linolenic acid metabolism, implying that coordinated interactions between microtubule dynamics and hormone signaling may underlie the disrupted epidermal architecture and aberrant surface patterning observed in rugose fruits (Figure 5d). In total, 9 microtubule-related and 23 phytohormone-related genes were identified (Figure S4; Tables S4 and S5), highlighting their interconnected roles in microtubule organization, hormone signaling, and cell division during rugose surface development.

To validate the transcriptome data, ten DEGs with relatively high expression levels were selected from a set of 32 microtubule- and phytohormone-related candidates for quantitative real-time PCR (qRT-PCR) analysis. The selected genes included *CA01g03980*, *CA01g06530*, *CA03g35180*, *CA03g35550*, *CA04g13900*, *CA05g16320*, *CA08g09800*, *CA08g17680*, *CA10g18960*, and *CA12g00700*. A strong positive correlation was observed between RNA-Seq and qRT-PCR results, with a Pearson correlation coefficient (R) > 0.8 (Figure S5). Although minor variations in absolute expression values were noted between the two methods, the overall expression trends were consistent, thereby confirming the reliability and reproducibility of the RNA-Seq data generated in this study.

### 2.5. Expression Profiling of Candidate Genes Across Different Tissues and Developmental Stages

**Figure 6:**
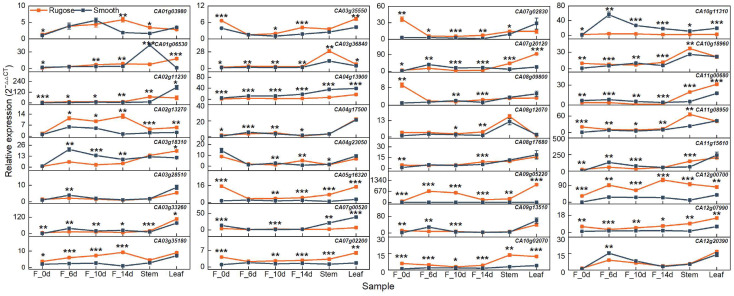
Expression profiles of 32 candidate genes across different tissues and developmental stages. The pepper *UBIQUITIN* (*UBI*) gene was used as a constitutive control. The relative expression was calculated using the 2^−ΔΔCt^ method. Orange lines represent the rugose surface variety, and blue lines represent the smooth surface variety. Data are shown as the mean ± SE from three technical replicates and three biological replicates. Statistical significance was determined by Student’s *t*-test: * *p* < 0.05; ** *p* < 0.01; and *** *p* < 0.001. F_0d, ovary; F_6d, fruit at 6 days post-anthesis; F_10d, fruit at 10 days post-anthesis; F_14d, fruit at 14 days post-anthesis.

To elucidate the functional roles of candidate genes potentially involved in rugose fruit formation, their expression patterns were examined across multiple tissues and developmental stages in the two parental lines exhibiting distinct epidermal textures. In addition to fruit samples collected at 6, 10, and 14 DPA (F_6d, F_10d, and F_14d), ovaries at anthesis (F_0d), stems, and leaves were also included for expression analysis. To refine the candidate gene list, priority was given to genes that (i) exhibited differential expression specifically in fruit between the two lines and (ii) showed pronounced expression divergence, particularly at stages coinciding with the onset of surface folding. Based on these criteria, ten genes were selected: five microtubule-related genes (*CA03g18310*, *CA03g28510*, *CA04g17500*, *CA09g13510*, and *CA12g20390*) and five phytohormone-related genes (*CA02g13270*, *CA03g35180*, *CA07g02830*, *CA08g12070*, and *CA10g11310*) (Figure 6). These genes exhibited significant expression differences between smooth and rugose fruits, with expression levels notably elevated in the rugose line, particularly at 10 and 14 DPA. These temporal expression patterns suggest that these genes may play stage-specific regulatory roles in cytoskeletal rearrangement, hormone response, and coordinated cell growth during morphogenesis of the fruit surface. Furthermore, the expression of several genes was preferentially enriched in fruit tissues rather than vegetative organs, reinforcing their potential specificity in fruit developmental regulation. These observations align with global transcriptome trends indicating enrichment of microtubule-related processes and hormone biosynthesis/signaling pathways. Collectively, the spatiotemporal expression characteristics of these candidate genes strongly support their functional relevance to the formation of rugose surface architecture in pepper fruits.

## 3. Discussion

The rugose surface trait in pepper, characterized by pronounced ridges and depressions, is associated with a crisper and more tender texture, which is generally favored by consumers. In the present study, it was demonstrated that the formation of rugosity in pepper affects fruit texture by modulating the activity of key enzymes involved in pectin metabolism (Figure 1). Histological analyses revealed that the development of the rugose surface was predominantly associated with a disorganized arrangement of epidermal cells, indicating that disturbances in epidermal cell alignment and structural integrity play a pivotal role in the manifestation of surface irregularities. Furthermore, the increased cell density per unit area observed in rugose fruits suggests elevated proliferative activity within the mesocarp. This enhanced cellular proliferation may lead to mechanical mismatches in tissue expansion between the epidermal and subepidermal layers, thereby facilitating the formation of surface folding. Similar coordination between division and expansion of mesocarp cells during early fruit development has been reported in tomato, where spatial–temporal variation in cell proliferation contributes to fruit morphology changes [39].

The coordinated development of multicellular tissues relies on the orderly division and expansion of distinct cell layers. In this study, histological analysis revealed that rugose fruits exhibit markedly disorganized epidermal cell arrangements compared to smooth fruits (Figure 2), suggesting that altered cell division orientations and polar hormone distribution may underlie the structural divergence [40,41]. In addition, impaired water transport and phloem flow may have inhibited vacuolar expansion, thus limiting cell enlargement and contributing to the formation of surface ridges [42].

Microstructural characteristics such as cell size, shape, and intercellular spacing are tightly associated with fruit texture, as they directly influence cell wall mechanical properties and tissue porosity [17,43]. Degradation of key cell wall components can weaken intercellular adhesion and disrupt pore architecture, leading to increased tissue softness and reduced mechanical resistance [44]. Generally, smaller, angular, and tightly packed cells are associated with a firmer texture, whereas larger, round, and loosely arranged cells result in a softer or mealy texture [45,46]. Therefore, the irregular epidermal cell structure observed in rugose peppers provides a key anatomical basis for the differences in fruit texture.

Developmental analysis revealed that the rugose phenotype gradually became apparent around 10 days post-anthesis (DPA), highlighting the dynamic nature of surface formation (Figure S6c). To investigate the molecular basis of this process, transcriptome profiling was conducted on fruits collected at three key developmental stages—pre-rugose (6 DPA), early-rugose (10 DPA), and post-rugose (14 DPA)—in both smooth- and rugose-fruited lines. DEGs were identified and subjected to K-means clustering and GO/KEGG enrichment analyses. These analyses revealed significant enrichment of microtubule-related and phytohormone-related genes among the DEGs (Figure 3 and Figure 5), indicating their central role in regulating surface patterning. Previous studies have shown that microtubule-related genes are essential components involved in cell division, cell expansion, and morphological development [47,48,49]. Among the microtubule-related genes, kinesins stood out as key regulatory elements (Table S4). Kinesins are essential motor proteins that generate forces along microtubule fibers through ATP hydrolysis, thus contributing to cellular morphogenesis and intracellular transport [50,51,52]. Several kinesin genes, including putative kinesin-13 homologs known to regulate microtubule dynamics and spindle assembly [53], showed higher expression in smooth fruits and decreased expression in rugose fruits as development progressed. This pattern suggests that kinesins may contribute to the maintenance of epidermal cell organization, and their downregulation may be linked to the disordered cell arrangements characteristic of rugose surfaces. Such structural disorganization may also stem from disruptions in cytoskeletal components such as tubulin, which is essential for maintaining directional cell expansion and tissue architecture [37,54]. Moreover, previous studies have demonstrated roles for kinesins in fruit shape regulation in cucumber and watermelon [55,56,57], further supporting their involvement in fruit surface morphogenesis.

In addition to cytoskeletal dynamics, plant hormones are critical regulators of cell expansion and division and, thus, play a fundamental role in shaping organ morphology [58,59,60,61]. This study focused on three phytohormone-related KEGG metabolic pathways potentially associated with rugosity formation—“plant hormone signal transduction”, “brassinosteroid (BR) biosynthesis”, and “(α-) linolenic acid metabolism”—all of which have been shown to regulate cell expansion and proliferation [62,63,64,65,66]. BRs are known to alleviate mechanical constraints by promoting loosening of cell wall structures, enabling coordinated tissue expansion [60]. Reduced BR biosynthesis in rugose fruits may increase epidermal stiffness, thereby imposing a mechanical constraint that disrupts the coordinated expansion between epidermal and subepidermal cell layers. This imbalance likely contributes to the rugose surface phenotype. In contrast, elevated BR levels in smooth fruits may promote uniform cell expansion, resulting in a smoother fruit surface. Additionally, α-linolenic acid metabolism contributes to the biosynthesis of jasmonic acid (JA) and methyl jasmonate (MeJA), which have been implicated in cell cycle regulation in other plant systems [67].

It is also plausible that ploidy variation within the fruit pericarp, as documented in other Solanaceae species, contributes to cell enlargement and structural asymmetry in rugose fruits [68,69]. Although their specific roles in fruit epidermal development remain unclear, the observed transcriptional differences suggest that altered phytohormone signaling may disrupt the spatial coordination of cell layer development, ultimately contributing to surface morphological divergence in pepper.

Through transcriptome analysis, five microtubule-related genes—encoding kinesin motor proteins—and five phytohormone-related genes were found to be significantly differentially expressed between smooth- and rugose-fruited lines (Figure 6). Recent studies have highlighted extensive crosstalk between kinesin-mediated microtubule dynamics and hormone signaling pathways in plant development [70,71,72]. Notably, *BHS1*, a kinesin-13a gene, functions as a negative regulator of BR signaling, thereby modulating plant architecture and grain size in rice [70]. These findings suggest that the kinesin and hormone-related genes identified in this study are strong candidates for regulating fruit surface morphology in pepper.

To further substantiate these findings, future research should incorporate cytological investigations (e.g., tubulin immunolabeling) and flow cytometry to assess ploidy variation. Functional validation of candidate genes will benefit from transgenic approaches, such as gene overexpression, targeted knockout, and complementation assays. In addition, integrative multi-omics strategies—encompassing transcriptomics, proteomics, and metabolomics—could facilitate the reconstruction of regulatory networks that govern epidermal patterning. Ultimately, elucidating the mechanistic interplay between microtubule dynamics and hormone signaling will be crucial for understanding the molecular basis of surface rugosity and texture formation in fleshy fruits such as pepper.

## 4. Materials and Methods

### 4.1. Plant Materials and Phenotype Observation

The F_2_ population used in this study was derived from a cross between two pepper inbred lines with contrasting fruit epidermal phenotypes. The female parent, 22Y5495, produces fruits with a markedly rugose surface characterized by epidermal ridges and depressions, whereas the male parent, 22Y5496, produces fruits with a smooth epidermis. These parental lines were selected based on preliminary sensory evaluations and quantitative assessments of texture-related attributes. The F_2_ population, consisting of 277 individuals, was used to investigate the relationship between rugosity index and texture quality. The plants were cultivated in a plastic greenhouse at the experimental stations of China Agriculture University in Sanya City, Hainan Province, China (18°25′ N, 109°51′ E). The development of pepper fruits from both parental lines were monitored from the ovary stage up to 30 days post-anthesis (DPA), with images captured at various developmental stages for subsequent analysis. Phenotypic evaluation was conducted for both fruit and vegetative organs. Plant height, leaf morphology, and stem development were visually inspected and photographed to assess potential systemic abnormalities.

At 30 DPA, fruits from the F_2_ population were harvested for phenotypic evaluation and texture measurements. For histological analysis, fruits from both parental lines were collected and processed for paraffin sectioning. Transcriptomic analysis was performed using samples from three key developmental stages: pre-rugose (6 DPA), early-rugose (10 DPA), and post-rugose (14 DPA) in both smooth- and rugose-fruited lines. Additionally, ovaries, stems, and leaves were collected from both parental lines for quantitative real-time PCR (qRT-PCR). A single fruit was taken from the mid-portion of each plant, and samples from five plants were pooled to constitute one biological replicate. Three biological replicates were prepared per time point. All samples were immediately flash-frozen in liquid nitrogen and stored at –80 °C until RNA extraction.

### 4.2. Development of Method for Phenotype Measurement of the F_2_ Population

The F_2_ population were classified into four levels for degree of rugosity using visual and tactile assessment: Level 1: smooth pericarp with no apparent roughness; Level 2: few surface protrusions with low roughness; Level 3: numerous surface protrusions with low roughness; and Level 4: numerous surface protrusions with high roughness.

To enable quantitative assessment of fruit surface rugosity, a CAD-based method was developed. A 3 cm longitudinal section was excised from the fruit equator and imaged alongside a scale reference. Using AutoCAD 2023 software, the outer epidermal contour was manually traced and converted into a polyline. The total epidermal length was calculated using the “LIST” command. The rugosity index (RI) was defined as:

RI (%) = (Epidermal Length-Section Length)/Section Length × 100

Four slices per fruit were used, and the mean RI value was calculated as the final measurement (Figure S6a,b).

### 4.3. Measurement of Biochemical Indicators Related to Texture Quality

For texture and biochemical analyses, thirty plants were randomly selected from the F_2_ population without consideration of molecular genotype or phenotypic extremes. These plants were not pre-classified or clustered based on parental similarity or genetic background. A 1 cm diameter cylindrical section was excised from the mid-region of each fruit using a sterile puncher. The remaining fruit tissues were immediately frozen in liquid nitrogen for subsequent determination of cell wall composition and pectinase activity. Texture parameters, including rupture force, hardness, adhesiveness, chewiness, and shear force, were measured using a texture analyzer (FTC, TMS-Touch) equipped with a 6 cm diameter cylindrical probe and a TMS lightweight blade set, following the manufacturer’s protocols.

Cell wall materials (CWMs), including water-soluble pectin (WSP), ionically bound pectin (ISP), covalently bound pectin (CSP), cellulose, and hemicellulose, were extracted following the modified protocols of Deng et al. [73] and Lin et al. [74]. Absorbance was measured at 530 nm after carbazole–sulfuric acid color development, and pectin content was expressed as mg·g^−1^ using galacturonic acid as a standard. A calibration curve was obtained using galacturonic acid as a standard. The results were expressed as mg·g^−1^. The cellulose content was determined by the gravimetric method [4]. The hemicellulose fractions were quantified by the anthrone–sulfuric acid assay using glucose as a standard.

The activities of polygalacturonase (PG), cellulose (CX), β-galactosidase (β-Gal), and pectin methyl esterase (PME) were determined. We added 5 mL of acetate–sodium acetate buffer (pH 5.5) containing 1 mmol/L Cys, 1 mol/L NaCl, and 1% PVPP to 1 g of the sample. We homogenized the mixture and incubated it at 4 °C for 3 h. We centrifuged the homogenate at 3500 r/min for 30 min at 4 °C and collected the supernatant as the enzyme extract. The enzymatic extracts (100 μL) were incubated for 1 h at 37 °C in 1 mL of 50 mmol/L acetate–sodium acetate buffer (pH 5.5) and 1 mL of 10 g/L polygalacturonic acid for PG enzymes. In another test tubes, 100 μL of the enzyme extracts was incubated for 1 h at 37 °C in a 1.5 mL of 10 g/L CX for CX enzymes. After being supplemented with 1.5 mL of DNS reagent, the mixture was boiled for 5 min. After cooling to room temperature, the volume was made to 25 mL with water and the absorbance at 540 nm was determined using glucose as a standard. The β-galactosidase activity was determined using the 4-nitrophenyl β-D-glucopyranoside (PNPG) as a substrate. The assays were performed in 2.5 mL of reaction mixtures containing 0.5 mL of enzyme extracts and 1 mL of PNPG dissolved in 50 mM NaOAc/HOAc buffer (pH 5.5). The reaction mixture was then incubated at 37 °C for 30 min. The reaction was terminated by adding 2 mL of 50 mM Na_2_CO_3_. The volume was increased to 25 mL with water, and the absorbance at 400 nm was determined using p-nitrophenol as a standard. PME activity was assayed according to Huang et al. [75] in a reaction mixture with 0.5% (*w*/*v*) pectin, 0.01% bromothymol blue, and enzyme extracts, recorded at 620 nm.

### 4.4. Paraffin Section and Scanning Electron Microscopy

The middle portion of each fruit were immediately excised and fixed with 50% FAA solution (50% ethanol, 5% acetic acid, and 3.7% formaldehyde) and 2.5% glutaraldehyde at 4 °C for approximately 24 h. Fixed samples with FAA solution were sequentially dehydrated in an ethanol series (70%, 75%, 85%, 95%, and 100%) and then in a graded xylene/ethanol series, with samples held in each solution for 1 h. Following dehydration, samples were embedded in paraffin according to Liu et al. [76]. The paraffin blocks were sectioned into 8 μm thick slices along the vertical axis, and the sections were stained with Safranin O-Fast Green Staining to observe under a microscope (N-800M, Novel Optical Instruments Co., Ltd., Ningbo, China). Fixed samples with glutaraldehyde were processed as previously described [77] and observed using JCM-7000 SEM with a 15 kV accelerating voltage.

### 4.5. RNA-Seq Analysis

Total RNA was extracted from pepper fruit tissues using the SteadyPure Plant RNA Extraction Kit (AG21019, Accurate Biotechnology, Changsha, China), and RNA integrity was assessed using the Agilent 2100 Bioanalyzer. mRNA was purified using the NEBNext Poly(A) mRNA Magnetic Isolation Module, and cDNA libraries were constructed with the NEBNext Ultra RNA Library Prep Kit following the manufacturer’s instructions. Sequencing was performed on the Illumina HiSeq 2000 platform.

Raw sequencing data were processed to remove adapters and low-quality reads (Q < 20), yielding clean reads. These were mapped to the *Capsicum annuum* CM334 reference genome using HISAT2, and gene expression levels were quantified as fragments per kilobase per million reads mapped (FPKM).

DEGs were identified with edgeR_DESeq2 based on an absolute Fold Change ≥ 2 and False Discovery Rate (FDR) < 0.01. Functional annotation of DEGs was conducted by BLAST (v2.13.0) searches against multiple databases, including the NCBI non-redundant protein (Nr), non-redundant nucleotide (Nt), and Swiss-Prot databases. Gene Ontology (GO) terms were assigned using the Blast2GO pipeline, and KEGG pathway enrichment analysis was performed with custom Perl scripts. GO enrichment was further refined using the “topGO” (v2.52.0) R package.

### 4.6. Expression Analysis Using qRT-PCR

Reverse transcription was performed using 1 μg of total RNA with the EvoM-MLV RT kit (AG11705, Accurate Biotechnology, China), which includes a gDNA removal step. Quantitative real-time PCR (qRT-PCR) was carried out in 10 μL reaction volumes containing 5 μL of 2× SYBR Green Pro Taq HS Premix (AG11735, Accurate Biotechnology), 1 μL of diluted cDNA, 0.2 μL each of forward and reverse primers (10 μM), and 3.6 μL of RNase-free water. Reactions were performed in 384-well plates on a qPCR platform (QuantStudio 6 Flex, Thermo Fisher Scientific, Waltham, MA, USA). The thermal cycling conditions were as follows: 95 °C for 30 s; followed by 40 cycles of 95 °C for 10 s and 60 °C for 30 s. *UBIQUITIN* (*UBI*) was used as the internal control gene. Relative expression levels were calculated using the 2^−ΔΔCt^ method [78]. The primer sequences used in this study are listed in Table S6.

### 4.7. Statistical Analysis

To evaluate the feasibility of the CAD-based rugosity quantification method, a linear regression analysis was performed to examine the relationship between the quantitative RI and the sensory grading results. The regression model was formulated as:

Y = β_0_ + β_1_ X + ϵ

where Y represents the sensory classification level, X denotes the CAD-derived RI, β_0_ is the intercept, β_1_ is the regression coefficient, and ϵ is the error term. Linear regression analysis was conducted using Origin (v.2025).

Statistical comparisons among multiple groups were performed using one-way ANOVA followed by Tukey’s honestly significant difference (HSD) test. Pairwise comparisons between two groups were analyzed using Student’s *t*-test. All statistical analyses were carried out in SPSS 22.0 (IBM SPSS Inc., Chicago, IL, USA), and *p* < 0.05 was considered statistically significant.

Pearson correlation coefficients among phenotypic and biochemical indicators were calculated using the R package corrplot (v4.1.0), and the results were visualized as a correlation heatmap.

## 5. Conclusions

This study elucidates the cellular and molecular basis underlying the rugose surface trait in pepper, a fruit quality attribute associated with enhanced crispness and consumer preference. Through an integrated analysis of fruit texture, histology, and transcriptomics, we demonstrate that surface rugosity is driven by epidermal cell disorganization, accelerated pectin degradation, and cell wall loosening. Key candidate genes involved in microtubule organization and phytohormone signaling were identified as major contributors to epidermal morphogenesis during critical developmental stages (10–14 DPA). These genes exhibited spatiotemporally regulated expression patterns that coincide with the emergence of surface folding, highlighting their functional relevance. Importantly, our findings not only deepen the understanding of fruit surface patterning in Solanaceae species but also establish a robust foundation for future molecular breeding strategies aimed at improving fruit texture. The identified candidate genes serve as promising targets for genetic manipulation to enhance fruit sensory quality, storage performance, and market value. Looking forward, further functional validation of these genes and their regulatory networks will enable precision breeding for desirable surface traits in pepper and potentially other horticultural crops. This work paves the way for integrating epidermal morphology as a key trait in pepper breeding programs.

## Data Availability

The raw RNA-Seq sequence data were deposited in the Short Read Archive (SRA) of the National Center for Biotechnology (NCBI) and are available under accession number PRJNA1279266.

## Supplementary Materials

The following supporting information can be downloaded at: https://www.mdpi.com/article/10.3390/plants14152451/s1, Figure S1: Correlation between sensory and CAD-based rugosity evaluation; Figure S2: Morphological comparison of various organs between the rugose-fruited line “22Y5495” and the smooth-fruited line “22Y5496”; Figure S3: Gene expression patterns during the development of pepper fruits; Figure S4: Trends in the changes in the expression of key genes associated with plant hormones in rugose surface and smooth surface pepper fruits; Figure S5: Validation of RNA-Seq data using qRT-PCR analysis; Figure S6: Schematic representation of the degree of rugosity surface evaluation method, with cross-sectional views and growth dynamics of the bumpy surface parent; Table S1: Summary of RNA-Seq sequencing data results; Table S2: Summary of RNA-Seq read mapping results; Table S3: Expression information for K-means clustering of 5019 differentially expressed genes (DEGs) into 16 co-expression modules; Table S4: Annotation information for 9 microtubule-related differentially expressed genes (DEGs); Table S5: Annotation information for 23 phytohormone-related differentially expressed genes (DEGs); Table S6: Primers used for qRT-PCR analyses.

## Abbreviations

The following abbreviations are used in this manuscript:

RI	Rugosity index
DPA	Days post-anthesis
TPA	Texture profile analysis
SEM	Scanning electron microscope
CWM	Cell wall materials
DMSO	Dimethyl sulfoxide
SF	Shear force
RF	Rupture force
HD	Hardness
AD	Adhesiveness
CH	Chewiness
HEM	Hemicellulose
CEL	Cellulose
WSP	Water-soluble pectin
ISP	Ionic-soluble pectin
CSP	Covalently soluble pectin
CX	Cellulose
PME	Pectin methylesterase
β-Gal	β-Galactosidase
PG	Polygalacturonase
CC	Cellular component
MF	Molecular function
BP	Biological process
DEG	Differentially expressed gene
