# Supplementary material for: Histological and Transcriptomic Insights into Rugose Surface Formation in Pepper (Capsicum annuum L.) Fruit
Source: Plants (Basel). 2025 Aug 7;14(15):2451. doi: 10.3390/plants14152451 (PMC12349362; doi:10.3390/plants14152451)
Supplement: Supplementary file 1 [file plants-14-02451-s001.zip › Supplementary Figure.pdf]

Supplementary Figures:
Histological and Transcriptomic Insights into Rugose Surface Formation in Pepper (*Capsicum annuum* L.) Fruit

Yiqi Xie [1,2], Haizhou Zhang [1,2], Chengshuang Li [1,2], Qing Cheng [1,2], Liang Sun [1,2,*] and Huolin Shen [1,2,*]

1 Department of Vegetable Science, College of Horticulture, China Agricultural University, Beijing 100193, China; xieyiqi277@163.com (Y.X.); zhz1999123@163.com (H.Z.); 13309045006@163.com (C.L.); chengqing2020@cau.edu.cn (Q.C.)

2 Sanya Institute, China Agricultural University, Sanya 572025, China

* Correspondence: shl1606@cau.edu.cn (H.S.); liang_sun@cau.edu.cn (L.S.)

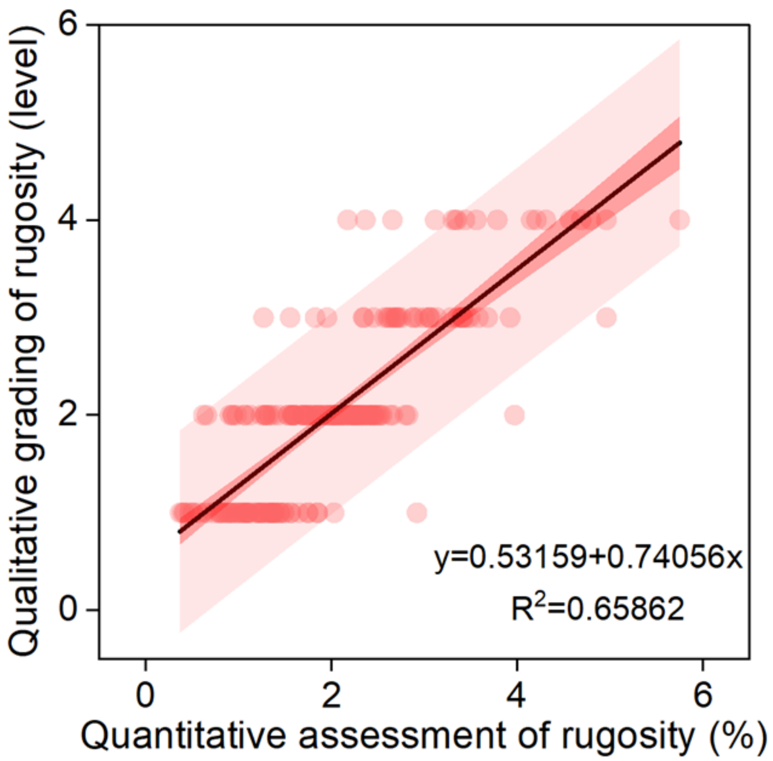

Supplementary Figure S1. Correlation between sensory and CAD-based rugosity evaluation.

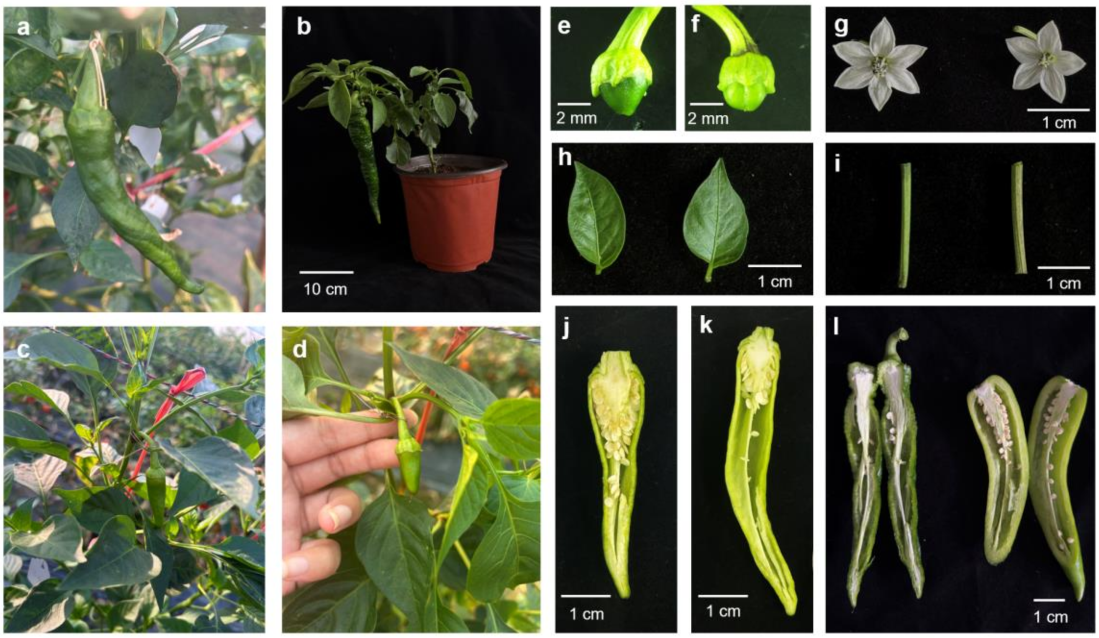

Supplementary Figure S2. Morphological comparison of various organs between the rugose-fruited line '22Y5495' and the smooth-fruited line '22Y5496'. (a–b) Whole-plant morphology of the rugose line '22Y5495'. (c–d) Whole-plant morphology of the smooth line '22Y5496'. (e–f) Ovary morphology at anthesis in rugose (e) and smooth (f) lines. (g) Flower of the rugose (left) and smooth (right) lines. (h) Leaf of the rugose (left) and smooth (right) lines. (i) Stem segments from the rugose (left) and smooth (right) lines. (j–k) Fruit longitudinal sections at 10 days post-anthesis (DPA) from the rugose (j) and smooth (k) lines. (l) Longitudinal sections of fruits at 30 DPA from the rugose (left) and smooth (right) lines.

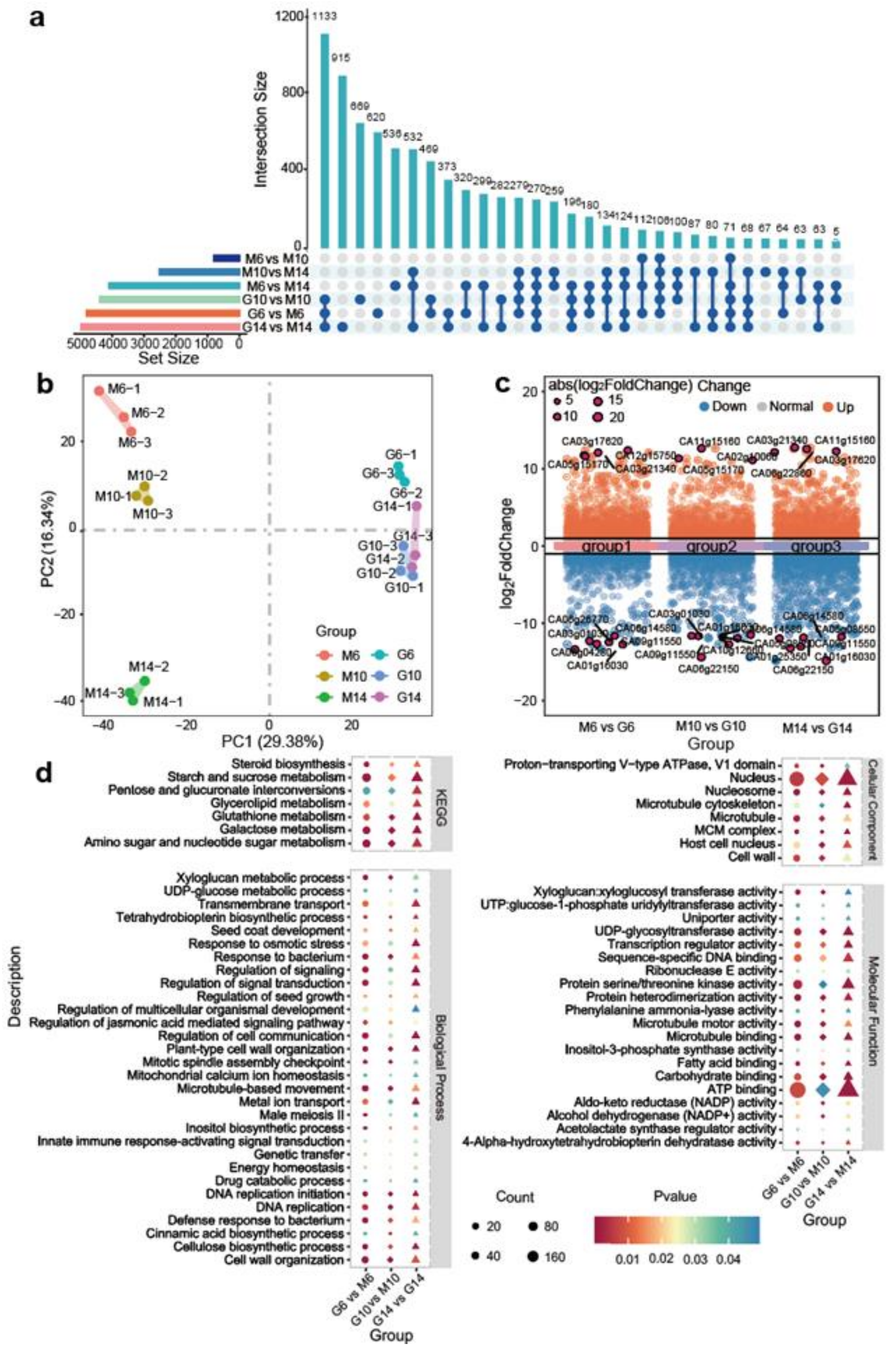

Supplementary Figure S3. Gene expression patterns during the development of pepper fruits. (a) Upset plot of genes identified in different development stages the comparison groups, Horizontal bar on left represents number of genes identified in each group. Dots and lines represent subsets of genes. Vertical histogram represents number of genes in each subset. (b) PCA (Principal component analysis) score plot of all DEGs in different comparison groups, PC1 and PC2 are two principal components. (c) Volcano plots show the differences in gene expression between different developmental stages of rugose surface and smooth surface pepper fruits. Orange, and blue dots indicate upregulated, downregulated genes. (d) KEGG pathway and GO enrichment analysis of DEGs for G6 vs M6, G10 vs M10, and G14 vs M14. The color of the point represents the P value, and the size of the point represents the number of enriched DEGs.

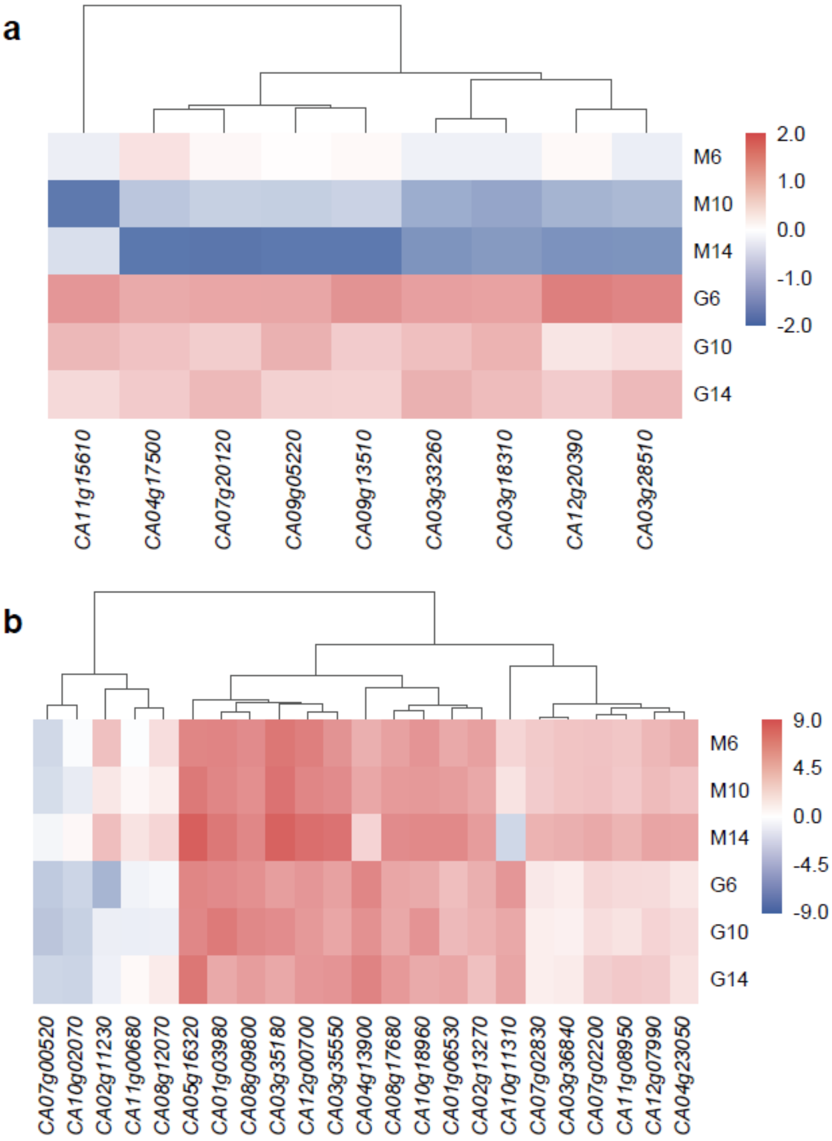

Supplementary Figure S4. Trends in the changes in expression of key genes associated with plant hormones in rugose surface and smooth surface pepper fruits. (a) Heatmap of differentially expressed genes related to microtubules identified through GO enrichment analysis. (b) Heatmap of differentially expressed genes involved in plant hormone signaling and key phytohormones metabolic pathways identified through KEGG enrichment analysis. The heatmap is drawn using the FPKM values of the transcripts in the transcriptome database. Each row represents a sample, and each column represents a gene. The color in the figure represents the gene's expression value after normalization in each sample; red indicates higher expression in that sample, blue indicates lower expression The up side shows a tree diagram of gene clustering and the down side shows gene names; the closer two gene branches are to each other, the closer their expressions are.

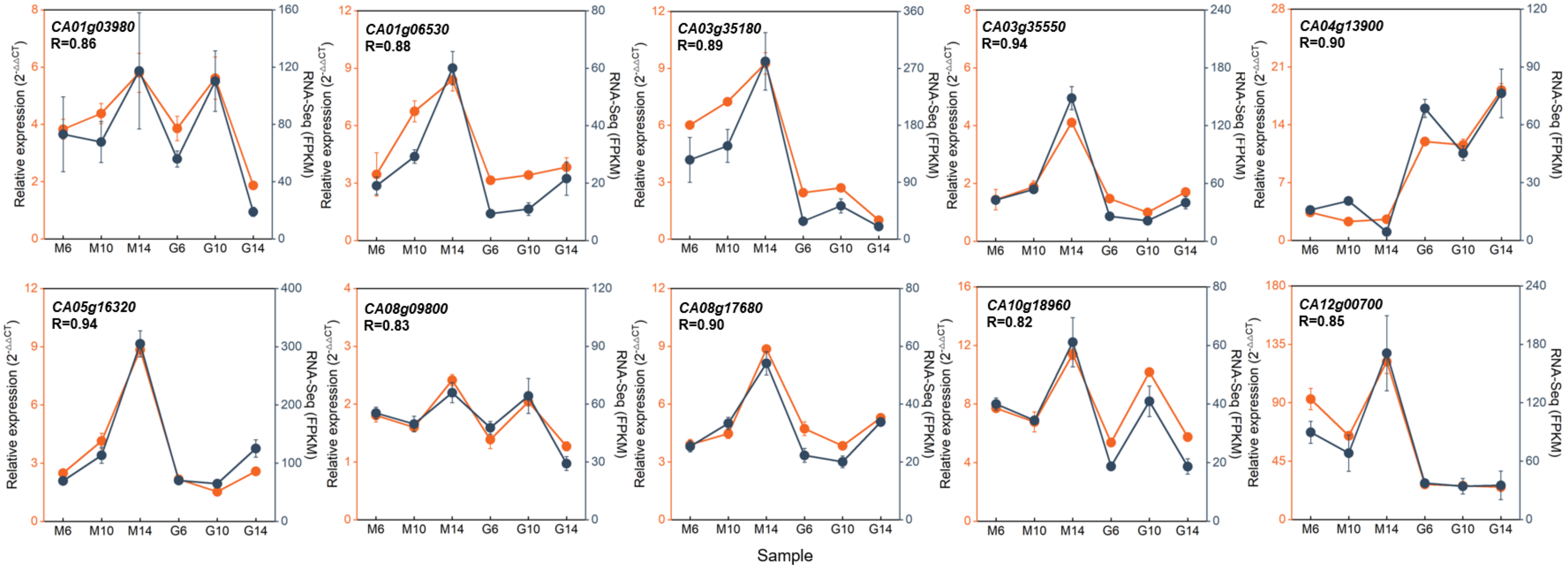

Supplementary Figure S5. Validation of RNA-Seq data using qRT-PCR analysis. A total of 10 DEGs were used for validation. The pepper *UBIQUITIN* (*UBI*) gene was used as a constitutive control. The relative expression was calculated using the $2^{-\Delta\Delta CT}$ method. Correlation analysis showed the correlation between RNA-Seq data and qRT-PCR was strong (Pearson's correlation coefficient (R) > 0.8). The left vertical axis refers to the quantitative real-time polymerase chain reaction (qRT-PCR, orange lines) and the right vertical axis indicates Fragments Per Kilobase of transcript per Million mapped reads (FPKM, blue lines). Data are presented as mean ± SE.

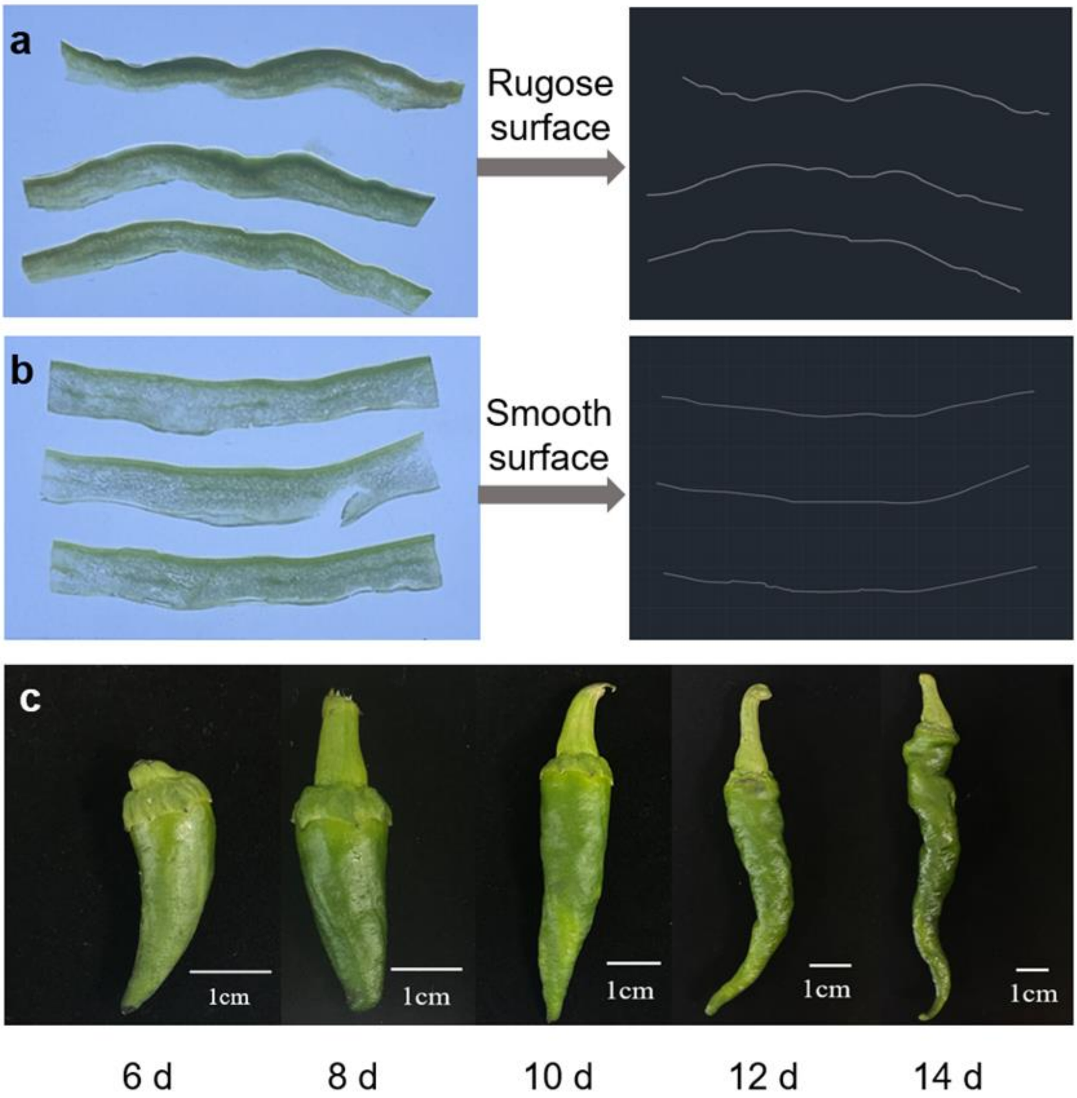

Supplementary Figure S6. Schematic representation of the degree of rugosity surface evaluation method with cross-sectional views (a-b) and growth dynamics of the rugose surface parent (c).
